# Food and human gut as reservoirs of transferable antibiotic resistance encoding genes

**DOI:** 10.3389/fmicb.2013.00173

**Published:** 2013-06-24

**Authors:** Jean-Marc Rolain

**Affiliations:** Unité de Recherche sur les Maladies Infectieuses et Tropicales Émergentes, UM63, CNRS 7278, IRD 198, INSERM 1095, Institut Hospitalo-Universitaire Méditerranée Infection, Aix-Marseille UniversitéMarseille, France

**Keywords:** antibiotic resistance, multidrug resistant bacteria, resistome

## Abstract

The increase and spread of antibiotic resistance (AR) over the past decade in human pathogens has become a worldwide health concern. Recent genomic and metagenomic studies in humans, animals, in food and in the environment have led to the discovery of a huge reservoir of AR genes called the resistome that could be mobilized and transferred from these sources to human pathogens. AR is a natural phenomenon developed by bacteria to protect antibiotic-producing bacteria from their own products and also to increase their survival in highly competitive microbial environments. Although antibiotics are used extensively in humans and animals, there is also considerable usage of antibiotics in agriculture, especially in animal feeds and aquaculture. The aim of this review is to give an overview of the sources of AR and the use of antibiotics in these reservoirs as selectors for emergence of AR bacteria in humans via the food chain.

## INTRODUCTION

The recent increase and spread of antibiotic resistance (AR) in human pathogens over the past decade has become a global problem, and is mainly due to the overuse and/or misuse of antibiotics in humans. However, there are many other sources of selective pressure such as use of antibiotics in agriculture that are known to be drivers of AR. Moreover, recent genomic and metagenomic studies in humans, animals, and the environment have led to the discovery of a large reservoir of new and/or unknown AR genes in these reservoirs that represent the so-called “ intrinsic resistome” that represent a large subset of non-acquired AR genes whose functions are multiple and complex in nature (Rolain et al., 2012; Baquero et al., 2013). Antibiotics are natural, semi-synthetic, or synthetic compounds with antimicrobial activity and are administered to both humans and animals for several purposes, including treatment, prevention, and growth promotion (animals). The mechanisms of intrinsic or “ natural AR” itself are complex and are of ancient origin and are marked by recent evolutionary adaptations. Intrinsic AR is a natural phenomenon developed by bacteria to protect antibiotic-producing bacteria from their own products and also to increase their chances of survival in highly competitive environments (D’Costa et al., 2011). Moreover, antibiotics produced by natural organisms have many other functions in nature mainly for cell to cell signaling networks and homeostasis of bacterial communities (Martinez and Rojo, 2011; Baquero et al., 2013). Thus production of antibiotics in environment is counterbalanced by natural resistant organisms in a socially cohesive ecological population (Cordero et al., 2012). Emergence of AR genes in pathogenic bacteria in humans is a recent phenomenon that occurred via mobilization from such AR gene pool (D’Costa et al., 2006; Forsberg et al., 2012) either through chromosomal mutations or mainly by lateral transfer of AR determinants from other bacteria. Although most of the AR genes found in human pathogens have a recent origin, there are pieces of evidence that AR genes could be also found in samples from pre-antibiotic era (D’Costa et al., 2011; Bhullar et al., 2012). Finally, because most of AR genes are located in nature in the “ intrinsic resistome” with many different functions, emergence of AR genes in clinical isolates represent only a small part of the total of these genes. Industrial use and release of antibiotics in microbial environments is a recent phenomenon with many consequences on bacterial population and evolution of bacteria in natural environments. It has been well-demonstrated that the use of antibiotics as growth promoters in food animals selects for AR bacteria that may spread from food to humans leading to human infection (Phillips et al., 2004). While there is extensive use of antibiotics in humans and food animals, there is also significant usage of antibiotics in agriculture, especially in animal feeds and in aquaculture. Finally, recent metagenomic studies have demonstrated that native human gut microbiota contain many bacteria that possess several AR-encoding genes that could be laterally transferred in the gut to potentially pathogenic bacteria (Scott, 2002; Salyers et al., 2004; Kazimierczak and Scott, 2007). Consequently, AR genes exist not only in nature and in humans but also in food animals, fish, plants, and vegetables as an enormous reservoir of genes that can be transferred to human pathogens either by direct contact or indirectly through the consumption of contaminated foods. The aim of this review is to give an overview of the reservoirs of these contemporary AR genes that were recently discovered in normal human gut microbiota, food animals, and agriculturally produced foods; to summarize the scientific evidence of the transfer of AR genes of bacteria from these pools; and to explore the emergence of AR bacteria in humans via the food chain.

## THE USE OF ANTIBIOTICS IN FOOD ANIMALS AND AQUACULTURE AND THE SUBSEQUENT EMERGENCE OF ANTIBIOTIC-RESISTANT BACTERIA

Antibiotics are used in food animals and aquaculture as therapeutic agents, prophylactics, and growth promoters (Phillips et al., 2004). For more than 50 years antibiotics used as growth promoters were thought to have only the positive effect of promoting growth in food animals, but the efficacy of growth promotion of these antibiotics were never rigorously tested (Graham et al., 2007). A recent large-scale economic analysis conducted in the United States has demonstrated that the use of antibiotics as growth promoters in food animal production is associated with economic losses for producers (Graham et al., 2007). Moreover, the overuse of antibiotics in food-source animal production has been shown to increase the risk of AR bacterial transfer to humans, and the use of most antibiotics for growth promotion has been banned by the European Union (EU) in 1999 (Casewell et al., 2003) following numerous studies that have linked the decrease of AR prevalence to the ban of antibiotic use in food animal production (Marshall and Levy, 2011). However, in some countries, such as China and United States, antibiotics as growth promoters and therapeutics in animals are not monitored despite a high occurrence of AR bacteria (Zhu et al., 2013). The main problem with the use of antibiotics in food animal production and aquaculture is that many of the antibiotics that are used are from antibiotic classes that are also used in the treatment of human bacterial infections. Thus, the use of one specific antibiotic as a growth promoter may also lead to cross-resistance with antibiotics used in medicine. Even more alarming is the possibility that the use of one antibiotic could select for multiple ARs to functionally unrelated antibiotics because AR genes could be associated with transferable plasmids and transposons (Marshall and Levy, 2011). Although several antibiotics have been withdrawn from use as growth promoters in animal feed production, many others still continue to be used for growth promotion and for the treatment of animals. An emerging field where antibiotics are given for prophylaxis is industrial fish aquaculture, especially shrimp, trout, and salmon (Cabello, 2006). This has resulted in an increase of both AR bacteria in fish pathogens and in AR in the environment (Cabello, 2006). The exchange of AR genes between aquatic environments and terrestrial environments by lateral gene transfer (LGT) occurs via transformation (uptake of free DNA), conjugation (DNA transfer between two bacteria via conjugative plasmids and transposons), conjugative transposition, and transduction (DNA transfer via a bacteriophage) events (Cabello, 2006). Thus, resistant bacteria from foods (animals, fish, plants, and vegetables) that are consumed by humans, as well as direct contact between humans and animals, water, fish, or vegetables, can be a source of AR gene transfer (Schjorring and Krogfelt, 2011).

## SOURCES OF ANTIBIOTIC RESISTANCE IN HUMAN PATHOGENS

### ANTIBIOTIC RESISTANCE IN FOOD ANIMALS

Antimicrobial agents administered to animals either for prophylaxis (growth promoters) or for therapy have been known to select for AR bacteria that could be transferred to human pathogens since 1975 with the first demonstration of the use of oxytetracycline as a growth promoter in chickens, which demonstrated an increased selection of tetracycline-resistant *Escherichia coli* colonization in the chickens, as well as the acquisition of such resistance in *E. coli* in the gut of the farm family (Levy et al., 1976a,b). This was studied during the previous decades to show the relationships between antibiotic usage as growth promoters in animals and the emergence of AR bacteria in animal and human infections (Marshall and Levy, 2011). Bacterial cross-resistance between antibiotics used in animal feed production and those used in humans has been clearly demonstrated by the use of avoparcin and the subsequent emergence and spread of vancomycin-resistant enterococci (VRE) as a dramatically life-threatening human pathogen (Hammerum et al., 2010). Avoparcin was banned in the EU in 1997 for prophylactic use, which led to a subsequent decrease in the carriage of glycopeptide-resistant *Enterococcus faecium* (Kazimierczak and Scott, 2007). This decrease was not observed in Denmark where the persistence of *vanA* genes in pigs was likely due to the use of tylosin (macrolide antibiotic) because the *ermB* and *vanA* genes were found to be encoded on the same mobile genetic element (Aarestrup, 2000). A short list of antibiotics that are used in animal food production and their structurally related antibiotics in human medicine is provided in **Table 1.** Thus, it is well established that AR bacteria that are selected in chickens, pigs, and cattle may be transmitted to the human intestine via the food chain (Salyers et al., 2004). The role of pets and wild animals as reservoirs of AR genes has been also documented (Costa et al., 2008; Poeta et al., 2009). Moreover, other compounds such as metals and biocides widely used in husbandry and veterinary can co-select AR genes such as glycopeptide resistance and macrolide resistance located in a transposon in *Enterococcus faecium* isolates from pigs in Denmark (Hasman and Aarestrup, 2005).

**Table 1:** Antibiotics used in food animal production, antibiotic resistance genes families, and related antibiotics used in humans.

Antibiotic class	Antibiotic resistance genes (*number of variants*	Name of antibiotic	Animal use	Related antibiotic in humans
Penicillins	*blaampC (111)*, *blaCTX (124)*, *blaOXA (204)*, *blaOXY (25)*, *blaSHV (102)*, *blaTEM (150)*	Amoxicillin, ampicillin	Treatment of swine and bovine	Amoxicillin, other penicillins
Glycopeptides	*van* gene clusters *(7)*	Ardacin*, avoparcin*	GP (bovine)	Teicoplanin, vancomycin
Polypeptide	NA	Colistin	GP (poultry)	Colistin, polymyxin B
Macrolides	*car (2)*, *cfr (2)*, *erm (30)*, *ole (2)*, *srm*, *tlr (2)*	Carbomycin, erythromycin, oleandomycin, spiramycin*, tylosin*	GP (poultry, swine) and treatment	Erythromycin, other macrolides
Streptogramins	*vga (4)*, *vat (7)*	Virginiamycin*	GP (poultry, swine)	Quinupristin+dalfopristin
Fluoroquinolones	*qnr (60)*, *qep (2)*, *nor*	Enrofloxacin, flumequine	Treatment	All quinolones
Aminoglycosides	*aac (67)*, *aad (28)*, *aph(32)*, *str(2)*	Gentamicin, neomycin, streptomycin	GP (poultry, swine) and treatment	All aminoglycosides
Sulfonamides	*sul (3)*	Sulfamerazine, sulfadimethoxine, sulfamethazine	GP (swine, chicken) and treatment	All sulfonamides
Tetracyclines	*tet (44)*	Oxytetracycline*, chlortetracycline*	GP (poultry, swine) and treatment	All tetracyclines

GP, growth promoter; EU, European Union; NA, not available.^*^Banned for growth promotion in the EU.

Recent metagenomic studies have revealed that AR genes are numerous in wild animals and food animals, and some recent examples are provided below. The effects of antibiotic exposure (amoxicillin, oxytetracycline and enrofloxacin) and the development of AR in *E. coli* from the chicken gut has revealed that even short-term exposure can lead to resistant strains (van der Horst et al., 2013). AR genes also increased in abundance and diversity in the intestinal microbiota of swine that received chlortetracycline, sulfamethazine, and penicillin, including resistance to antibiotics that were not administered (i.e., aminoglycoside *O*-phosphotransferases), which demonstrated the potential collateral effects of indirect selection of resistance to other classes of antibiotics (Looft et al., 2012).

The effects of penicillin supplementation in the feed of broiler chickens was recently assessed using the 454 pyrosequencing method and revealed an elevated percentage of Firmicutes and a decreased proportion of Bacteroidetes, which was similar to what was reported in human obese versus lean individuals (Singh et al., 2013). Similarly, it has been recently shown using functional metagenomic screening that long-term treatment of adult honeybees with oxytetracycline for control of larval pathogens is associated with a high incidence of tetracycline resistance (Tian et al., 2012). Recently, using high-capacity quantitative PCR targeting 244 AR genes, Zhu et al. (2013) showed that the reservoir of AR genes in large-scale commercial swine farms in China was enormous by detecting 149 unique AR genes from different classes of antibiotics of human medical interest. Moreover, along with the detection of numerous AR genes, they found a large enrichment in transposase sequences with some of them involved in mobilization of AR genes (Aziz et al., 2010), suggesting an increased risk of LGT of AR genes from livestock animals to human pathogens (Zhu et al., 2013). Similar findings regarding swine as reservoirs of AR genes have also been reported in the United States (Whitehead and Cotta, 2013). Finally, other foods may contain AR genes that are present in lactic acid bacteria and bifidobacteria, including milk and dairy products, which have been extensively studied and reviewed elsewhere (Ammor et al., 2007,^2008^). Foodborne urinary tract infections (FUTIs) has been recently revisited as a new paradigm for antimicrobial-resistant foodborne illness (Nordstrom et al., 2013). One of the most impressive examples of food as a source of antibiotic-resistant bacteria in humans is –the multidrug resistant extraintestinal pathogenic *E. coli* (ExPEC) that is responsible for community-acquired urinary tract infections (UTIs; Vincent et al., 2010) including the outbreak of trimethoprim–sulfamethoxazole-resistant *E. coli* UTIs in women from the United States (Manges et al., 2001), a pyelonephritis outbreak (Johnson et al., 2002), and the community outbreak of clonally related extended-spectrum beta-lactamases (ESBLs) type CTX -M infections worldwide (Pitout et al., 2005; Pitout and Laupland, 2008). The source of the strains responsible for these outbreaks has been linked to contaminated meat and other foods suggesting that the use of antimicrobials in food animal production may select for antibiotic-resistant strains of ExPEC. Moreover, a high prevalence of contamination by antimicrobial-resistant and ExPEC in retail foods has been reported, especially in turkey products purchased in grocery stores in retail markets from the United States (Johnson et al., 2005a). There are other examples of foods as reservoirs for AR bacteria that could be transmitted to humans via the food chain. Johnson et al. (2005b) have reported that retail foods may be a source for community transmission of AR ExPEC, which are now recognized as clinically significant foodborne pathogens. This has also been reported in Canada in retail chicken, meat, and pork (Manges et al., 2007; Vincent et al., 2010). AR clinical isolates of *E. coli* that were indistinguishable from chicken isolates have also been reported in Barcelona, Spain and in Minnesota and Wisconsin, the United States, suggesting that foodborne transmission of AR ExPEC is a very common phenomenon and is a major source of AR genes transmissible to humans via the food chain (Johnson et al., 2006,^2007^). A novel mosaic tetracycline resistance-encoding gene, tet(S/M), in a transposon likely acquired from *Lactococcus lactis*, has been described in foodborne strains of *Streptococcus bovis*, a human opportunistic pathogen (Barile et al., 2012).

### ANTIBIOTIC RESISTANCE IN AQUACULTURE

Aquaculture is a relatively recent and growing field where antibiotics are heavily applied, either directly to the water or in fish food, as prophylactics to control infectious diseases, which has resulted in the emergence of AR gene reservoirs in fish and aquatic animals as well as in the aquatic environment (Heuer et al., 2009). Different classes of antibiotics are used, including some that are critically important in human medicine, such as aminopenicillins, macrolides, aminoglycosides, fluoroquinolones, and tetracyclines (Heuer et al., 2009). The indirect spread of AR genes by LGT from antibiotic-resistant bacteria in fish to human pathogens has been widely documented on *Aeromonas* strains of fish origin that could easily transfer their AR plasmids to human pathogens, such as *Aeromonas hydrophila*, *E. coli*, and *Salmonella* (Cabello, 2006; Heuer et al., 2009). Conversely, transferable multidrug resistant plasmid from *Salmonella enterica* to *Aeromonas salmonicida* subsp. *salmonicida* has been also reported (McIntosh et al., 2008). Yet another example is the emergence of multidrug resistant strains of *Vibrio cholerae* that were responsible for the Latin American outbreak in 1992, which was likely transmitted by water and seafood (Weber et al., 1994). This was also demonstrated in multidrug resistant *Vibrio parahaemolyticus* and *Vibrio alginolyticus* isolates from farmed fishes in Korea (Oh et al., 2011). Similarly, AR genes found in the emerging human pathogen *Salmonella enterica* serotype Typhimurium DT104, responsible for outbreaks of salmonellosis in humans in Europe and in the United States, likely originated from an aquaculture setting (Cabello, 2006). An AR gene cluster of *Salmonella* Typhimurium DT104 containing a florfenicol resistance-encoding gene, which is an antibiotic widely used in aquaculture, was detected in these human isolates (Briggs and Fratamico, 1999). A transferable multiple antibiotic and mercury resistance plasmid was also reported in Atlantic Canadian isolates of *Aeromonas salmonicida* subsp. *salmonicida* (McIntosh et al., 2008). Thus, there is ample evidence that many AR determinants found in pathogenic human bacteria have a fish origin because these determinants could be detected in fish pathogens (Cabello, 2006). Direct transmission of AR bacteria from aquatic environments, resulting in human infection, has also been reported in cases involving direct contact with water and aquatic environments, drinking water or the consumption of contaminated fish products (Heuer et al., 2009). Although fish and aquaculture environments could be a reservoir of AR genes, marine sediment and oceans also have a wide pool of AR genes (ocean resistome; Yang et al., 2013). A recent metagenomic study using a high-throughput sequencing approach reveals that marine sediments in China contain numerous putative AR genes to 11 different classes of antibiotics of medical importance (especially genes conferring resistance to aminoglycosides, glycopeptides, and bacitracin), including sequences that were highly similar to transposons and plasmids found in human pathogens, suggesting that marine sediment bacteria play a major role in LGT of AR genes (Yang et al., 2013). Moreover, in the latter study, authors were able to identify sequences of a transposon similar to a 5.5-kb mega element that contains macrolide resistance genes. Although these macrolide resistance genes could be located in other genetic platforms, this mega element is the most prevalent molecular mechanism of macrolide resistance in *Streptococcus pneumoniae* human infections in the United States, Canada, and the UK (Zhanel et al., 2006). Examples of AR genes detected in aquaculture over the last 5 years are summarized in **Table 2**. Besides the use of antibiotics, there is also a risk of AR transmission by lactic acid bacteria used as probiotics in aquaculture (Muñoz-Atienza et al., 2013).

**Table 2:** Molecular detection of AR genes studies in aquaculture during the last 5 years of each species.

Samples	Type of study	Country	Bacterial species	Number of samples	AR gene detected*	Reference
Retail markets	Culture	China	*Aeromonas* spp.,Enterobacteriaceae	500**	*sul1*; *sul2*; *bla*_TEM;_ *bla*_CMY;_ *ermB*;*ermC*	Ye et al. (2013)
Marine sediments	Metagenomic	China	Proteobacteria, Firmicutes,Actinobacteria	NA	Aminoglycosides, tetracycline, chloramphenicol, beta-lactams, glycopeptides, fluoroquinolone, bacitracin, macrolide, streptogramin, sulfonamide	Yang et al. (2013)
Carp farms	Culture	Pakistan,Tanzania	*Acinetobacter baumannii*, *Aeromonas hydrophila*, *Alcaligenes*, *Enterobacter cloacae*, *Pseudomonas*, *Enterococcus casseliflavus*, *Kurthia*, *Proteus*, *Providencia*, *Vagococcus*	253	*sul1*; *sul2*; *dfrA1, 5, 7, 12, 15*; *bla*_TEM_; *cat-1*;*mefA*;*tetA*	Shah et al. (2012)
Farm fish	Culture	China	*Escherichia coli*	218	*bla*_CTX-M-14, M-79_; *bla*_SHV-27_; *qnrB*,*qnrS*, *qnrD*, *aac(6^′^*)-Ib-cr	Jiang et al. (2012)
Fish farm	Culture	Italy	Enterococci	650	*tetM*, *msrC*, *blaZ*	Di Cesare et al. (2012)
Fish farm	Culture	Japan	*Photobacterium damselae*	1	*bla*_CARB-9-like_, *floR*; *mphA-like*; *mefA-like*; *sul2*; *tetM*; *tetB* on a plasmid	Nonaka et al. (2012)
Fish farms	Culture	China	All genera	NA	*sul1*; *sul2*;*sul3*;*tetB*; *tetO*; *tetQ*; *tetT*; *tetW*; *tetM*	Gao et al. (2012)
Fish farms	Culture	China	Enterobacteriaceae	203	*tetA*; *tetC*; *sul2*; *aadA5*;*aadA22*, *dfr2*, *dfrA17*	Su et al. (2011)
Trout farms	Culture	Australia	*Aeromonas*	90	*aadA2*; *tetA*; *tetC*; *sul1*;*strA-strB*	Ndi and Barton (2011)
Aquaculture sediments	Molecular detection	Wisconsin (USA)	Not available	4 facilities	*tetR* genes	Seyfried et al. (2010)
Ornamental fish water	Culture	UK, Singapore, Guyana, Colombia	*Aeromonas*		*bla*_TEM-1_; *tetD*; *tetE*; *qnrS*; *dfr12*; *sul1*; *floR*; *catB8*; *bla{*_OXA7_; *arr2*; *aac(6′*)-Ib; *aadA2*	Verner-Jeffreys et al. (2009)
Salmon farms	Culture	Chile	*Pseudomonas*	119	*floR*	Fernandez-Alarcon et al. (2010)
Water fish aquaculture	Culture	China	*Vibrio*, *Pseudoalteromonas*	66	*cat*; *floR*; *tetB*, *tetD*; *tetE*; *tetM*	Dang et al. (2009)
Water from shrimp and fish ponds	Culture	Vietnam	*Acinetobacter*, *Aeromonas*, *Escherichia coli*; *Bacillus*; *Pseudoalteromonas*; *Shigella*	86	*sul1*; *sul2*; *sul2* on plasmids	Phuong Hoa et al.(2008)

*Names of AR-encoding genes are given when available.

### ANTIBIOTIC RESISTANCE IN RAW FRUITS AND FRESH VEGETABLES

Vegetables could also be a source of AR genes via contaminated raw fruits and fresh vegetables that are not sufficiently treated with water before consumption (Ruimy et al., 2010a). For example, *Pseudomonas aeruginosa* strains resistant to various classes of antibiotics, including ampicillin, chloramphenicol, and sulfamethoxazole/trimethoprim, have been isolated from the following foods/locations: fresh lettuce, carrots, tomatoes, and cucumbers from markets in Jamaica (Allydice-Francis and Brown, 2012), raw salads contaminated with tetracycline and trimethoprim/sulfamethoxazole-resistant *Shigella* species in Tunisia (Mokhtari et al., 2012) and fresh lettuce from markets in two cities of Mexico contaminated with AR *Salmonella* species (Castaneda-Ramirez et al., 2011). Interestingly, in the study from Tunisia, the authors reported a seasonal isolation of *Shigella* species from human and food samples during summer, suggesting that some human invasive infections could be linked to seasonal vegetable consumption. Seasonal bacterial infections have recently been reported in *Klebsiella pneumoniae* bloodstream infections on four continents (Anderson et al., 2008). ESBLs of the CTX-M-15 family, an ESBL encoding gene endemic worldwide in *E. coli* human infections, was detected in a *Pseudomonas teessidea* strain cultured from a prepackaged retail spinach as well as a novel ESBL of the bla-RAHN-2 family from *Rahnella aquatilis*, suggesting that saprophytes in common fresh vegetables may be an important source of AR genes that could spread laterally to human pathogens (Raphael et al., 2011). The ESBLs of the RAHN family have been described in *R. aquatilis* strains that were isolated from raw fruits and vegetables (Ruimy et al., 2010a,b). Traditionally fermented foods of animal and vegetable origins may also contain AR bacteria, such as *Enterococcus faecium* and *Enterococcus faecalis*, both of which exhibit a high incidence of resistance to rifampicin, ciprofloxacin, and quinupristin/dalfopristin, and surprisingly, vancomycin (Sánchez et al., 2013). Finally, the recent significant outbreak of hemolytic uremic syndrome in Germany in 2011 was found to be due to a CTX-M-15 ESBL *E. coli* clone likely originating from fenugreek seeds imported from Egypt (Buchholz et al., 2011; Rasko et al., 2011; Weiser et al., 2013).

## NORMAL HUMAN GUT MICROBIOTA AS A RESERVOIR FOR AR GENES

Thanks to the use of new technologies, such as high-throughput pyrosequencing, metagenomic studies and exhaustive culture techniques, a description of human microbiota has begun to revolutionize our current understanding of the relationship between gut microbiota composition, several diseases and obesity (Lagier et al., 2012). It is now accepted that numerous factors, including age, geographical provenance and environment, dietary habits, antibiotics and probiotics, can modify the composition of the human gut microbiota (Lagier et al., 2012). Thus, it is not surprising that the human gut microbiota represents an important reservoir of AR genes present in the bacterial community and that these AR genes may be transferred from one bacterium to another bacterium in the gut, especially human pathogens (Salyers et al., 2004). Although AR in commensal *E. coli* from healthy people was reported more than 40 years ago (Smith and Halls, 1966), recent studies have highlighted an increase and high incidence of AR in commensal *E. coli* from both children and from adults (Bailey et al., 2010). However, there are few reports dealing with the identity of the AR genes carried by healthy humans. Several functional metagenomic studies have been successfully applied to human gut microbiota, including healthy adults or infant individuals, to discover the presence of a vast diversity of new AR-encoding genes that could be a source for the emergence of new AR genes in human pathogens in the future (Gueimonde et al., 2006; Sommer et al., 2009; Cheng et al., 2012). One of the most recent and surprising findings in humans was that approximately 2/3 of individuals living in a remote community in South America without any antibiotic selection pressure carried bacteria resistant to at least one antibiotic (Bartoloni et al., 2009). Another research area, especially during the past years, is studying the development of gut microbiota in infants to show that gut microbiota is an important reservoir of AR genes (Valles et al., 2012). Antibiotic-resistant bacteria responsible for invasive infections in children are increasing worldwide, and it has been shown using metagenomics that such AR bacteria and AR gene transfer (tetracycline resistance determinants) could be transmitted from mother to child and persist for weeks after birth (de Vries et al., 2011). Thus AR genes and bacteria could be transmitted vertically from the mother’s gastrointestinal tract, birth canal, or breast milk to the infant at a very early age. Moreover, the human gut is known to be a favorable environment for LGT (Palmer et al., 2007), and the spread/transfer of AR genes from non-pathogenic to pathogenic bacteria have been documented even in the case of young breast-fed children (de Vries et al., 2011). Tetracycline resistance-encoding genes (*tetA* and *tetB*) have also been detected in 12% of 309 commensal intestinal strains of *E. coli* from 128 Swedish infants who did not receive tetracyclines during their first year of life (Karami et al., 2006). A recent study in India revealed a high prevalence of multidrug resistant (MDR) and carbapenem-resistant *Acinetobacter baumannii* colonization from the gut of neonates, including one particular baby in which the same clone was found in both its blood and stool samples (Roy et al., 2010). Infant gut microbiota in Greece at days 4, 30, and 90 after delivery were found to contain as many as 34% of vancomycin-resistant *Lactobacillus* species (Kirtzalidou et al., 2011). Zhang et al. (2011) reported the presence of several AR genes including *tetM*, *sul2*, and *bla*_TEM_ in *Enterococcus*, *Staphylococcus*, *Klebsiella*, *Streptococcus*, and *Escherichia/Shigella* species from the gut microbiota of 16 American infants without antibiotic exposure who had fed on breast milk or infant formula from birth to 1-year-old. A recent study in Australian healthy adults has shown that commensal *E. coli* represent a huge reservoir of AR-encoding genes including genes conferring resistance to trimethoprim (*dfrA1*, *dfrA5*, *drfA7*, *dfrA12*, or *dfrA17*), sulfamethoxazole (*sul1*, *sul2*, or *sul3*), tetracycline (*tetA* or *tetB*), and ampicillin (bla_TEM_; Bailey et al., 2010). Similarly, bla_NDM-1_ human carriage cases have been reported in several countries in India, China, and Pakistan as well as in the environment (Nordmann et al., 2011).

Although antibiotic treatment in humans directly affects the pathogen responsible for the infectious disease, antibiotic use has been shown to lead to a collateral effect in commensal bacteria in the gut microbiota. In fact when AR genes are selected in the commensal gut microbiota, they could be further transferred to human pathogen. Karami et al. (2007) have shown that a plasmid-encoded beta-lactamase encoding gene of the TEM family from an ampicillin-resistant *E. coli* was transferable to a susceptible strain in the gut of a children treated with ampicillin for UTIs. It has been shown that the administration of antibiotics could alter the bacterial community structure of the gastrointestinal microbiota in mice and that these modifications are different according to the antibiotic classes administered (Robinson and Young, 2010). Moreover, these modifications are not transient only (Jakobsson et al., 2010) in human gut microbiota; recent data have been accumulated demonstrating that some classes of antibiotics may affect the global composition of the gut microbiota and the persistence of AR bacteria in the human intestine for years (Jernberg et al., 2010). It has been shown that the diversity of gut microbiota is reduced after antibiotic therapy in children (Penders et al., 2006) and in elderly patients (Bartosch et al., 2004,^2005^). Deep 16S rRNA pyrosequencing of the human gut microbiota from three healthy humans before and after treatment with ciprofloxacin has also shown a dramatic shift in bacterial abundance (Dethlefsen et al., 2008). Interestingly, it has been demonstrated that antibiotic treatment has a dramatic effect on bacterial diversity even after only 1 week of treatment and that AR, in this case the macrolide resistance gene *ermB*, can persist for long periods (4 years; Jakobsson et al., 2010). Broad-range culture techniques and metagenomic analyses have confirmed that antibiotic treatment, especially broad-spectrum antibiotic treatment, dramatically changes the gut microbiota in humans, which could lead to the emergence of invasive infections (Raum et al., 2008; Cotter et al., 2012; Perez-Cobas et al., 2012; Dubourg et al., 2013a,b). Löfmark et al. (2006) and Jernberg et al. (2007) have shown that 1 week of clindamycin administration could select for resistant *Bacteroides* spp., which could persist for as long as 2 years following clindamycin exposure. Antibiotic use could also lead to a collateral effect through the selection of AR bacteria among the normal microbiota, as has been demonstrated with the long-term persistence of clarithromycin-resistant *Enterococcus* species harboring the *ermB* gene immediately after treatment to eradicate *Helicobacter pylori*, which may persist for 1–3 years after antibiotic therapy (Sjolund et al., 2003). Similarly, Jakobsson et al. (2010) have shown that clarithromycin and metronidazole administration in patients suffering from peptic ulcer disease may select for the presence of the *ermB* gene, which may persist in some cases for up to 4 years post-treatment, demonstrating that the AR gene may be stabilized in the human gut microbiota following selection. Thus, the increased prevalence of AR and possibility LGT between bacteria are important consequences of the long-term persistence of AR genes in gut microbiota (Jernberg et al., 2010).

## CONCLUSION

While the overuse and misuse of antibiotics in humans is undoubtedly associated with the emergence and spread of AR bacteria and AR genes, at least in human infections, recent studies have demonstrated that the use of antibiotics in animals, aquaculture and most importantly, food production, is also a major factor contributing to the emergence of AR bacteria and the spread of AR genes. Moreover, because the potential pool of AR genes in these environments remains largely unknown, with thousands of AR genes yet to be discovered, future observation of AR in these ecosystems is warranted from an ecological perspective. This phenomenon is compounded by the immense number of methods that bacteria have developed for the exchange of AR genes via LGT, recombination, and transposition and also for the purpose of creating new AR genes, as recently exemplified with the discovery of the new chimeric carbapenemase encoding gene NDM-1 (Yong et al., 2009; Toleman et al., 2012). Further studies are warranted and should utilize a combination of culture and non-culture-based techniques to better characterize the resistome in both the human gut microbiota, in response to various conditions and diseases, and in non-human environments, including animals, aquaculture, and food products, to better understand the role of antibiotics in the evolution of these ecosystems.

## Conflict of Interest Statement

The author declares that the research was conducted in the absence of any commercial or financial relationships that could be construed as a potential conflict of interest.
